# Effect of 12-weeks elastic band resistance training on MyomiRs and osteoporosis markers in elderly women with Osteosarcopenic obesity: a randomized controlled trial

**DOI:** 10.1186/s12877-021-02374-9

**Published:** 2021-07-20

**Authors:** Ebrahim Banitalebi, Majid Mardaniyan Ghahfarrokhi, Mortaza Dehghan

**Affiliations:** 1 Department of Sport Sciences, Shahrekord University, Shahrekord, Iran; 2 Clinical Research Development Unit, Kashani Hospital, Shahrekord University of Medical Sciences, Shahrekord, Iran

**Keywords:** MyomiRs, Osteoporosis markers, Resistance training, Osteosarcopenic obesity

## Abstract

**Background:**

Interorgan communication networks established during exercise in several different tissues can be mediated by several exercise-induced factors. Therefore, the present study aimed to investigate the effects of resistance-type training using elastic band-induced changes of myomiRs (i.e., miR-206 and miR-133), vitamin D, CTX-I, ALP, and FRAX® score in elderly women with osteosarcopenic obesity (OSO).

**Methods:**

In this randomized controlled trial, 63 women (aged 65–80 years) with Osteosarcopenic Obesity were recruited and assessed, using a dual-energy X-ray absorptiometry instrument. The resistance-type training via elastic bands was further designed three times per week for 12-weeks. The main outcomes were Fracture Risk Assessment Tool score, bone mineral content, bone mineral density, vitamin D, alkaline phosphatase, C-terminal telopeptides of type I collagen, expression of miR-206 and miR-133.

**Results:**

There was no significant difference between the study groups in terms of the Fracture Risk Assessment Tool score (*p* = 0.067), vitamin D (*p* = 0.566), alkaline phosphatase (*p* = 0.334), C-terminal telopeptides of type I collagen (p = 0.067), microR-133 (*p* = 0.093) and miR-206 (*p* = 0.723).

**Conclusion:**

Overall, the results of this study illustrated 12-weeks of elastic band resistance training causes a slight and insignificant improvement in osteoporosis markers in women affected with Osteosarcopenic Obesity.

**Trial registration:**

Randomized controlled trial (RCT) (Iranian Registry of Clinical Trials, trial registration number: IRCT20180627040260N1.

Date of registration: 27/11/2018.

**Supplementary Information:**

The online version contains supplementary material available at 10.1186/s12877-021-02374-9.

## Background

Aging is associated with several changes in bones, muscles, and body fat percentage (BFP) due to decreased levels of anabolic steroids and sex hormones. The term osteosarcopenic obesity (OSO) has been recently proposed for the relationship between losses of muscle and bone mass and increased fat mass [1–3].

Both bone and skeletal muscle also share similar mesenchymal origins and respond to trophic effects of hormones, growth factors, inflammatory mediators, loading and exercise training [4], as well as communication via paracrine/endocrine factors [5]. Interorgan communication networks (ICNs) established during exercise in several different tissues can be mediated by some exercise-induced factors. Emerging evidence indicates that muscle-bone communication may be also achieved by the release of some myotube-derived exosomal microRNAs (myomiRs) to neighboring cells [6], and myomiRs can further transport health-promoting information of exercise interventions to other tissues in endocrine and paracrine manners [7] so that myomiRs can be released into the blood serum/plasma, wherein their levels are influenced by exercise and diseases [5, 8]. In this respect, An et al. had demonstrated that expression of some myomiRs (i.e., 133a, 206, and 204) had changed in the bone tissue of ovariectomized mice [9].

The potential mechanisms by which exercise can improve OSO syndrome-related parameters are multifactorial and may relate to the regulation of genes, circulating hormone levels, and metabolic pathways [10]. Considerable evidence exists that resistance training alone regulates energy balance, stimulates anabolic process, promotes muscular hypertrophy, reduces fat mass, and improves bone density [11] and resistance training may improve OSO phenotypes in older women [12]. It is assumed that skeletal muscles and bone tissues respond preferentially to mechanical loadings in an intensity-dependent manner [13]. Accordingly, the use of elastic bands is cheaper than traditional resistance training with weights having a significant effect on improving body composition, physical functions, physiological adaptations, and balance [14]. Unlike resistance machine-based training, a wide range of upper and lower body exercises can be easily performed in any location using such bands [15].

To the best of the authors’ knowledge, no study has thus fare evaluated the effects of elastic-band resistance training on myomiRs and some osteoporosis markers such as C-terminal telopeptides of type I collagen (CTX-I), Fracture Risk Assessment Tool (FRAX®) score, alkaline phosphatase (ALP), and vitamin D. Despite being theoretically clear, this molecular pathway-related myomiRs and osteoporosis communication have never been tested in response to resistance training. Considering this background, the present study aimed to determine the effects of resistance-type training using elastic band-induced changes of myomiRs (i.e., miR-206 and miR-133), vitamin D, CTX-I, ALP, and FRAX® score in elderly women with OSO. It is of note that circulating myomiRs have been recently identified as biomarkers for age-associated osteoporosis.

Based on the pivotal roles of circulating myomiRs in bone remodeling and their extracellular shuttling, it was first hypothesized that circulating myomiRs could have the potentials to show functional relevance to bone remolding [16]. Secondly, it was hypothesized that myomiRs released during exercise training could facilitate muscle-bone communication. It was further shown that expression of myomiRs could be directionally sensitive to exercise training. While the physiological function of such myomiR changes has not been well described, altered myomiR expression may govern long-term muscle growth. No research has been also reported in which myomiRs may be involved in osteoporosis and response to exercise training.

## Methods

### Design

This 12-week randomized controlled trial (RCT) (Iranian Registry of Clinical Trials, trial registration number: (IRCT20180627040260N1; https://www.irct.ir/trial/32463; Date of registration: 27/11/2018) was approved by the Iranian Ethics Committee of Sport Sciences Research Center (IR.SSRC.REC.1398.040). All the study participants also provided written informed consent.

### Cohort study

Based on the sample size used in previous research and along with comprehensive assessment protocols, the participants in this study were recruited via community-wide and general practitioner advertising in the city of Shahrekord, Iran. A detailed telephone screening process was thus conducted to identify those possibly excluded from the study. This was followed by assessment tests and all the participants underwent medical screening to confirm their eligibility based on the following inclusion criteria.

The participants were enrolled regarding to the Consolidated Standards of Reporting Trials (CONSORT) statement for randomized trials of non-pharmacologic treatments. The eligible participants, aged 65–80 years, were selected by a physician. Therefore, a total number of 102 women with OSO were recruited and assessed, using a dual-energy X-ray absorptiometry (DEXA) instrument. The inclusion criteria in this study were, age > 60–80 years, BFP > 32%, body mass index (BMI) > 30 kg/m2, − 2.5 ≤ T-score ≤ − 1.0 of L1-L4, and/or total femur (TF) or femoral neck (FN), gait speed (10-m walk test (10MWT)) ≤ 1 (m/s), and skeletal muscle mass index (SMI) ≤ 28% or ≤ 7.76 kg/m^2^ [3]. Moreover, not receiving hormonal therapies, participating in no regular exercise training> 30 min once a week over the last 6 months, taking no nutritional supplements within the past 3 months, and obtaining a Montreal Cognitive Assessment (MoCA) cut-off score ≥ 21 were among other criteria. The participants were excluded if they had resting blood pressure ≥ 160/100 mmHg, fasting triglyceride≥5.7 mmol/L, a history of cardiovascular diseases (CVDs), thyroid problems, cancer, endocrine disorders such as diabetes, kidney or liver diseases, surgeries, smoking, or use of recreational drugs or alcohol.

### The whole body composition scan

The regional body composition human body such as whole-body bone mass, soft tissue composition, fat mass normalized by height squared (FMI = Fat mass / Height^2^, appendicular lean mass index (ALMI = [arms + legs lean mass] / Height^2^), appendicular lean mass to BMI ratio (ALM/BMI), skeletal muscle mass percentage (%SMM), skeletal muscle mass (SMM), fat-free mass index (FFMI), fat mass index (FMI) and total body fat percentage (%BF) were performed using a whole-body Dual Energy X-ray Absorptiometry (DXA) scan by subdividing the body using specific well-defined cut lines [17–19].

### Sample size and test power

The sample size was calculated considering two-way repeated-measures analysis of variance (ANOVA) with two groups, type-I error = 5% and type-II error = 20%, statistical test power = 80%, and effect size (ES) = 0.20. The ES of the elastic band resistance training (EBRT) program was also estimated at 41 W for the FRAX®score. Considering these parameters as well as the use of G*Power software (Version 3.1.9.2), a total sample size of 52 individuals (26 cases per group) was determined. The sample size was consequently considered by 63 participants (experimental group, *n* = 32, and control group, *n* = 31) to accord with the anticipated 20% dropout rate.

### Randomization and concealment strategy

**Fig. 1:**
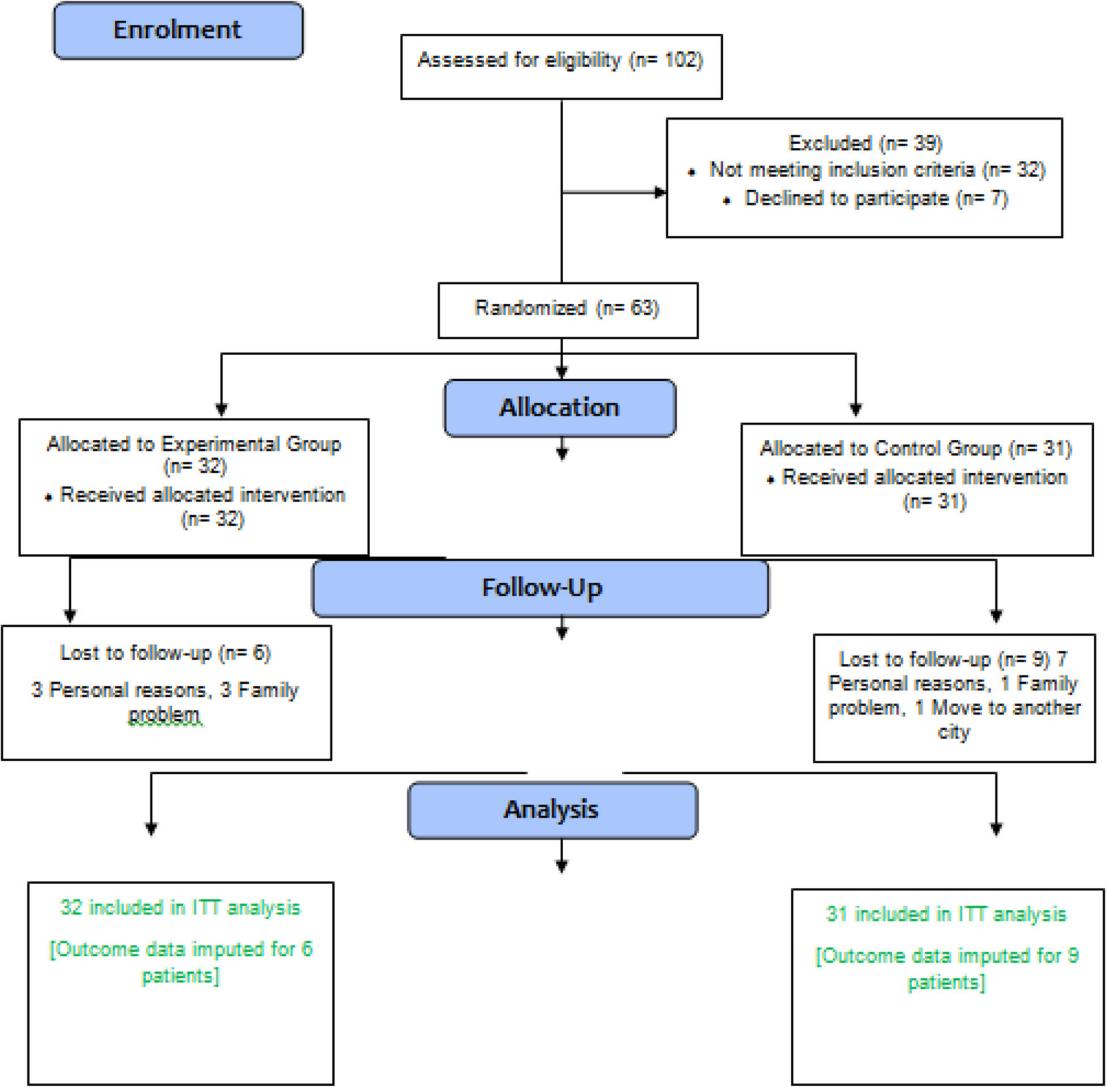
CONSORT flow diagram representing study design

The randomization was also fulfilled by an external researcher, not involved in testing or training programs, using randomly permuted block allocation with a block size of four. The participants were stratified according to two cut-off scores for each stratification of age (60–70 or 70–85 years) and OSO Z-score (− 3 to 0 or 0 to+ 3). The allocation was further concealed from those responsible for designing the exercise training protocol or monitoring the control group until the beginning of the training period. Neither participants nor researchers were blinded due to the nature of the intervention. Besides, exercise trainers, not involved in data collection, managed the exercise session program and monitored the individuals in the control group. The participants in the control group also received no diet intervention or changes in their typical diet or physical activity habits all through the study. Moreover, they received telephone calls or face-to-face interviews once a week to be assured that there had been no changes in their physical activity and diet habits during this study. Via weekly visits, health problems, functional problems, as well as medication use were recorded by a trained researcher. At the same time, the researchers reinforced the obligations to maintain their typical diet and activity habits. The participants were also randomly assigned to the experimental (namely, EBRT) group (*n* = 32) or control group (*n* = 31) (Fig. 1).

### Training protocol

The participants were instructed on how to use two exercise devices during the first two sessions before beginning the training protocol. In addition, they became familiar with the targeted number of repetitions (TNRs) and OMNI-resistance exercise scale (OMNI-RES) to control exercise intensity in the first two sessions [20]. The participants also had to increase or decrease grip width to adjust the resistance easier. Additionally, they were asked to choose an elastic band grip width, allowing them to perform 20 repetitions maximum (RM). The EBRT program (using Thera-Band®, the Hygienic Corp., Akron, OH, USA) was further designed to train all major muscle groups (namely, legs, back, abdomen, chest, shoulders, and arms). Training volume and intensity were also progressively increased and performed three times per week. Exercise training took place in small groups of not>10participants and was supervised by trained and experienced exercise physiologist. Each exercise session consisted of a general warm-up of 10 min, followed by a resistance training session (60 min) incorporating one to two exercises (in a slow controlled manner, 2 s for concentric phase, and4 s for eccentric phase), and was finally completed by a cool-down routine. Following an adaptation phase of 4weeks (1 set of 12 rep) using low resistance (yellow Thera-Band), exercise intensity progressively augmented by adapting the resistance of the elastic band (based on the Thera-Band® force-elongation table) from yellow to red and further to black. Additionally, the exercise volume was enhanced by adding to the number of sets from one to two. Progression rate was also based on individual improvements (band color was changed if the participants would have been able to perform two more repetitions in the second set and reported to be below seven on the OMNI-impulse response (OMNI-IR) for active muscle scale (0: extremely easy to 10: extremely hard) [20] (Supplementary Table S1 and S2). The participants in the control group also received telephone contacts or face-to-face interviews on a weekly basis to maintain their typical diet and activity habits.

### Adverse events

All the defined adverse events that occurred during or up to 48 h after resistance training were recorded every session and reported to the local Ethics Committee.

### Measurements

All pre- and post-measurements of the experiment were conducted by the same assessor blinded to treatment allocation. Assessments were further performed at baseline and 48 h after the last session in both groups. Demographic characteristics and medical history information were also collected through questionnaires.

Firstly, CTX-I (Cat number: EKU03502, sensitivity 52.9 pg/ml) in fasting serum was evaluated via the commercial enzyme-linked immunosorbent assay (ELISA) kits. To measure the serum levels of ALP for bone, kits from Pars Azmoon Co.(Iran) were used employing a synthetic photometric method (U/L measurement unit). Besides, vitamin D levels were measured employing an ELISA kit. All the evaluations were accordingly performed before and 48 h after the last training session (12 weeks of exercise intervention exercise) in both training and control groups. Serum miRNA was then extracted using the mirVana PARIS Ambion kit, followed by real-time polymerase chain reaction (RT-PCR). The U6 gene was additionally recruited as a housekeeping one.

Blood samples were collected in the fasting condition at the baseline and 48 h after the last session in fasting status. All the reagents were also prepared at room temperature and under accordance with the manufacturer’s protocols. Total RNA including messenger RNA (mRNA) and miRNA was isolated from the serum with RNX-Plus solution kit (Fermentase, Cinagen Co., Iran) and miR-amp kit (Pars genome Co., Iran) respectively under the manufacturer’s protocols (using chloroform layer separation followed by treatment with isopropanol and ethanol).

The total RNA (10–5 μg) or mRNA (10–500 ng) was reversely transcribed into cDNA. RT-PCR assay was further conducted using SYBR Green RT-PCR Master Mix kit (Applied Biosystems) for the quantification of mRNA. Expressions of miRNA and mRNAs were accordingly normalized to the β-Actin housekeeping gene. The relative amount of mRNA for each target gene was calculated based on its threshold cycle (Ct) compared with the Ct of the housekeeping (i.e. reference) gene (i.e., glyceraldehyde 3-phosphate dehydrogenase: GAPDH). The relative quantification was further performed by the 2^ (^∆∆Ct^) method.

A validated and calibrated FRAX® tool for Iran, based on the individual analysis of each patient, was used to evaluate the fracture risks of the participants. This algorithm could calculate the probability of a broken bone from clinically easy factors, resulting in the possibility of a fracture of the femur or other bones in the next ten years. This probability could be further calculated from data such as age, gender, BMI, family history of the bone, smoking, long-term use of steroids, rheumatoid arthritis (RA), and high alcohol consumption.

### Statistical analyses

Data analysis strategy was chosen regardless of intervention adherence level. The assumption of data normality was also checked using the Kolmogorov-Smirnov test before conducting the parametric tests. Descriptive data also included means, standard deviations (SDs), and percentage distributions. An independent-sample t-test was correspondingly used for baseline comparisons. A two-way repeated-measures ANOVA was also employed to determine the main changes (two times× two groups) after 12 weeks of training. Besides, Bonferroni’s method was applied wherein a significant interaction effect was observed. Partial eta-squared (ηp2) was additionally used to determine ES in the ANOVA. Additionally, the Pearson correlation coefficient was applied to investigate the correlation between myomiRs and osteoporosis markers, and the statistical significance was set at *p* < 0.05. Furthermore, intention-to-treat (ITT) analysis was performed at all stages of data analysis in this RCT. The data were analyzed using the IBM SPSS Statistics software (Version 22.0) for Windows (SPSS Inc., Chicago, IL, USA) and then expressed as mean ± SD.

## Results

The recruitment process during this pilot period can be found in the CONSORT flow chart in Fig. 1. Of the 102 participants screened, 63 met the inclusion criteria. The data from 29.03% (*n* = 9) and 18.75% (*n* = 6) of the subjects respectively from the control and training groups who did not attend the post-test measurements were then excluded. Subsequently, outcome data for 15 participants were included in the ITT. The main reasons for dropping out of the study were personal problems, unwillingness, and moving to another city. The rate of adherence to training sessions was also 85% in the experimental group. No more significant side effects were reported by researchers who were not blinded during the 12-week intervention than the group assignment. Mean and standard deviation of participant characteristics at baseline presented in Table 1.

**Table 1:** Study characteristics by groups at baseline

Variable	Control (***n*** = 31) Mean ± SD	Experimenta (***n*** = 32) Mean ± SD
Age *(years)*	64.05 ± 3.35	64.11 ± 3.81
Height *(cm)*	155.77 ± 4.14	155.59 ± 4.38
Weight *(kg)*	78.73 ± 7.52	81.81 ± 8.03
BMI *(kg/m*^*2*^*)*	32.53 ± 2.01	33.72 ± 3.15
Body fat (*%)*	43.60 ± 2.66	46.29 ± 3.42
BMC *(gr)*	2.13 ± 0.50	2.24 ± 0.38
BMD *(gr/cm*^*2*^*)*	1.005 ± 0.450	0.929 ± 0.245
FRAX	4.72 ± 0.18	4.68 ± 0.19
Vitamin D	43.02 ± 20.97	38.28 ± 21.32
Alkaline phosphatase *(IU/L)*	139.23 ± 28.22	165.93 ± 42.46
CTX-I *(ng/ml)*	0.526 ± 0.097	0.543 ± 0.081

*BMI* body mass index, *BMC* bone mass content, *BMD* bone mass density, *FRAX* Fracture risk assessment tool, *CTX-I* C-telopeptides of type I collagen

### Anthropometric profile

Result showed no significant difference between the study groups in height (F = 0.019, *p* = 0.889, ES = 0.001), weight (F = 0.602, *p* = 0.440, ES = 0.007), BMI (F = 0.354, *p* = 0.553, ES = 0.004) and total fat percent (F = 2.888, p = 0.093, ES = 0.030) (Table 2).

**Table 2:** Anthropometric profile changes following elastic-band resistance training in study groups

Variable	Group	Mean (SD)		%∆	***P***-value	F,T	Effect Size
		Pre test	Post test				
Age *(years)*	Control (n = 31)	64.05 ± 3.35			0.947	−0.067	
	Experimental (*n* = 31)	64.11 ± 3.81					
Height *(cm)*	Control (*n* = 31)	155.77 ± 4.14	155.08 ± 4.59	−0.44	Group = 0.964 Time = 0.641 Group×time = 0.889	0.002 0.218 0.019	0.001 0.002 0.001
	Experimental (*n* = 31)	155.59 ± 4.38	156.15 ± 4.89	0.36			
Weight *(kg)*	Control (*n* = 31)	78.73 ± 7.52	81.66 ± 10.09	3.72	Group = 0.409 Time = 0.422 Group×time = 0.440	0.687 0.650 0.602	0.007 0.007 0.007
	Experimental (*n* = 31)	81.81 ± 8.03	81.87 ± 9.82	0.07			
BMI *(kg/m*^*2*^*)*	Control (*n* = 31)	32.53 ± 2.01	33.33 ± 4.05	0.73	Group = 0.317 Time = 0.614 Group×time = 0.553	1.012 0.256 0.354	0.011 0.003 0.004
	Experimental (*n* = 31)	33.72 ± 3.15	33.65 ± 3.67	−0.06			
Total fat (*%)*	Control (*n* = 31)	43.60 ± 2.66	47.60 ± 2.65	9.17	Group = 0.400 Time = 0.001 Group×time = 0.093	0.714 22.046 2.888	0.008 0.193 0.030
	Experimental (*n* = 31)	46.29 ± 3.42	47.35 ± 3.86	2.29			

*BMI* body mass index

### Osteoporosis markers

**Fig. 2:**
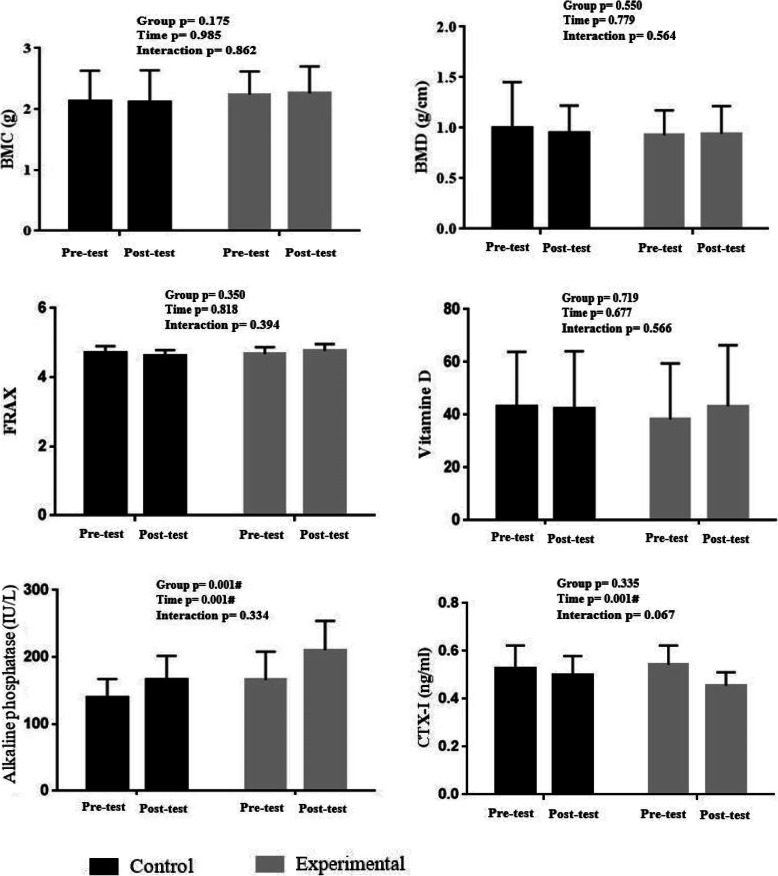
Osteoporosis markers change following 12 weeks of elastic band resistance training. BMD: bone mass density; BMC: body mass content; FRAX: fracture risk assessment tool; CTX-I: C-telopeptides of type I collagen. # indicates a significant difference at *P* < 0.05

The results of the two-way repeated measures ANOVA also showed no significant difference between the study groups with respect to BMC (F = 0.030, *p* = 0.862, ES = 0.001), BMD (F = 0.335, *p* = 0.564, ES = 0.004), and FRAX® score (F = 3.427, *p* = 0.067, ES = 0.036). No significant difference was further observed considering vitamin D (F = 0.333, p = 0.566, ES = 0.006), ALP (F = 0.945, p = 0.334, ES = 0.013), and CTX-I (F = 3.427, *p* = 0.067, ES = 0.036) (Fig. 2).

### MyomiRs

**Fig. 3:**
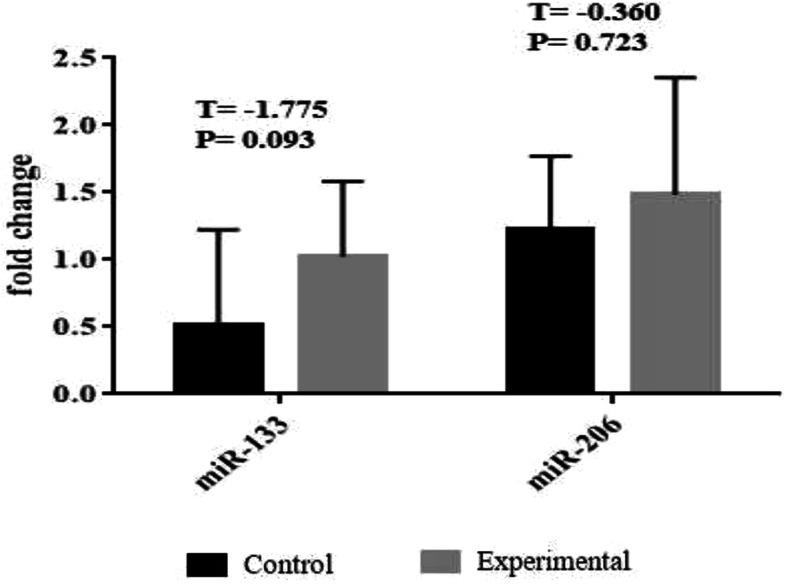
myoMirs (miR-133 and miR-206) following 12 weeks of elastic band resistance training

No significant difference was reported in miR-133 (F = -1.775, *p* = 0.093) and miR-206 (F = -0.360, *p* = 0.723) between the study groups (Fig. 3).

### Correlation between MyomiRs and osteoporosis markers

The results established no significant correlations between MyomiRs and Osteoporosis Markers at baseline measurements.

**Fig. 4:**
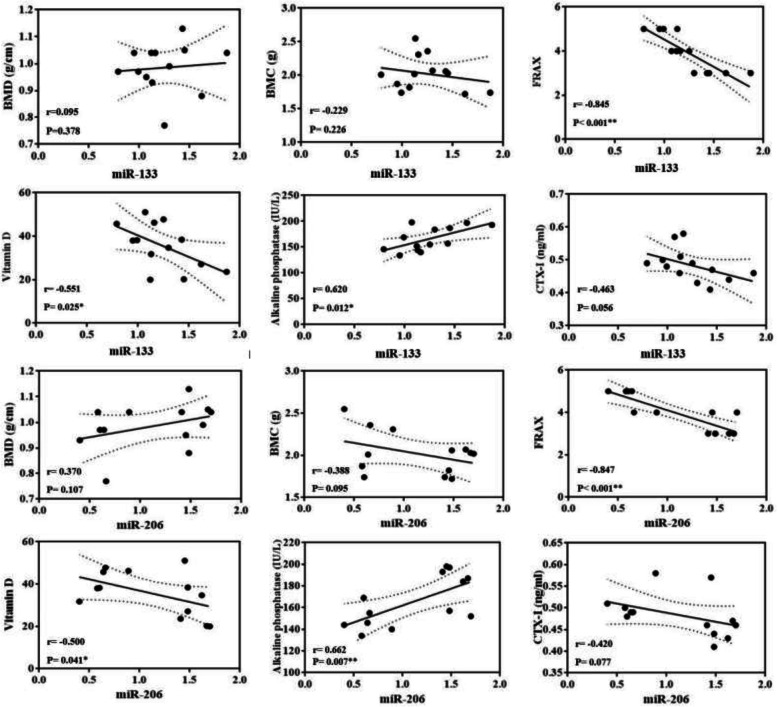
Bivariate correlation between myoMirs (miR-133 and miR-206) and osteoporosis markers (BMC, BMD, FRAX, vitamin D, alkaline phosphatase, and CTX-I) following elastic-band resistance training. BMD: bone mass density; BMC: bone mass content; FRAX: Fracture Risk Assessment Tool; CTX-I: C-telopeptides of type I collagen; miR-133: MicroRNA-133; miR-206: MicroRNA-206. * and ** indicates significant correlation at *P* < 0.05 and *P* < 0.01 respectively

Significant correlations were found between miR-133 and FRAX® score (r = − 0.845, *p* < 0.001), vitamin D (r = − 0.551, *p* = 0.025), and ALP (r = 0.620, *p* = 0.012), but not BMD (r = 0.095, *p* = 0.378), BMC (r = − 0.229, *p* = 0.226), and CTX-I (r = − 0.463, *p* = 0.056). There was also a significant relationship between miR-206 and FRAX® score (r = − 0.847, p < 0.001), vitamin D (r = − 0.500, *p* = 0.041), and ALP (r = 0.662, *p* = 0.007), but not BMD (r = 0.370, *p* = 0.107), BMC (r = − 0.388, *p* = 0.095), and CTX-I (r = − 0.420, *p* = 0.077) (Fig. 4).

## Discussion

Contrary to the research hypothesis addressed in this study, resistance-type exercise training with the elastic band did not have any effects on chronic levels of some serum myomiRs and osteoporesis markers in women with OSO syndrome. However, it was notable that the given changes in myomiRs were directly associated with variations in FRAX® score, serum vitamin D, and ALP concentrations, but not BMD, BMC, and serum CTX-I levels following resistance training.

Only a few studies so far have attempted the effects of exercise training on myomiRs and osteoporosis markers in elderly populations. However, to the best of our knowledge, this RCT was the first attempt to evaluat the effects of this type of exercise training on some myomiRs and osteoporosis markers in elderly women living with OSO syndrome. For example, Gombos et al. (2016), had compared resistance training with aerobic exercise, wherein CTX-I in the elderly women with very low bone density had significantly increased in the control group [21]. There seem to be two main reasons for the discrepancy between our results and the findings of the current study. First, in the study by Gombos et al., CTX-I levels had been measured immediately following a exercise training session, but measurements had been taken 48 h after the last exercise session in our study. Also, the very low levels of bone density in the study by Gombos et al. could be a reason for the increasing impact of exercise training intervention. On the other hand, Janik et al., (2018), in line with the present study, had revealed that 12 weeks of exercise had failed to have a significant effect on CTX-I levels of middle-aged and elderly women with osteoporosis syndrome [22]. They had further suggested that longer periods of exercise training modality would be needed to achieve better results. As well, Moazami and Jamali (2014), consistent with the results of the present study, had stated that 6 months of aerobic training could not significantly alter the levels of serum ALP in obese women [23]. Regardless of the significant difference between the two studies, aerobic exercise had not provided the necessary force on bone tissue for optimal metabolic changes. In agreement with the results of the present study, Gombos et al. (2016) had not observed a significant difference after 12 weeks of resistance training in serum ALP levels in elderly women [21]. Exercise alone had not thus induced significant changes in ALP levels, especially in elderly women diagnosed with osteoporosis. While in the study by Hassanzadeh et al. (2012), it had been reported that exercise combined with calcium and vitamin D supplementation had induced significant changes in serum.

ALP levels in postmenopausal women [24].

On the other hand, in the present study, serum levels of vitamin D did not change in a significant manner. One of the main reasons for no significant changes in bone turnover markers levels in the present study seemed to be the inadequacy of calcium and vitamin D consumption and even the diet of the participants [24], which could strongly affect the results. Moreover, there were no significant improvements in bone density indices (i.e., BMD and BMC), which in turn could influence bone markers.

Although myomiRs are emerging as potential key mediators of exercise adaptation in skeletal muscles, specific thresholds of intensity required for muscle-specific miRNA and upregulation of bone-specific markers, have not been rigorously assessed until now. Nevertheless, it was observed that expression of miR-206 and miR-133a, as well as bone turnover markers were not significantly different in the study groups following 12 weeks of resistance training. The very low responses of the myomiR and the osteoporosis markers to exercise training was not surprising.

However, there were some possible pieces of evidence and explanations. One plausible explanation was a short period of study, training intensity, and participant status. Another potential explanation could be associated with the selective release of these myomiRs by skeletal muscle and to serum in response to exercise training protocols [25]. Thus, unchanged expression of these miRNA in circulation following exercise training might be due in part to the limited release of miRNAs by skeletal muscle tissues. Thirdly, further analysis in the present study indicated that myomiRs might play a role in the phenotypic change of muscle and pronounced intergroup variations in exercise training responses [26]. These results did not correspond with the findings of the published studies in this area, indicating that some of these myomiRs [27, 28], had changed in response to exercise training regimes in skeletal muscle. Furthermore, Nielsen et al. had illustrated that the expression of miR-1, miR-133a, miR-133b, and miR-206 had decreased following 12 weeks of endurance training [27]. Other researchers had further confirmed that miRNA-133 [29, 30] and miRNA-206 [30, 31] had contributed to regulating obesity, and adipose tissue, regulation of osteoporosis and bone metabolism [32, 33], as well as muscle sarcopenia and atrophy [34, 35].

Another possible explanation was that exercise training modalities might mitigate old age-associated differences in miRNA expression in skeletal muscle. However, muscle contraction-induced adaptations in gene expression had differed between young and old populations [36, 37]. The altered gene expression pattern induced by both exercise training regimes with old age would not improve the miRNA expression within the skeletal muscle. Although it has been shown that muscle and bone are both mechanoresponsive tissues [38], it seems that younger individuals have more mechanoresponsiveness to myogenic and osteogenic signals, hormones, growth factors, and cytokines compared with older ones [39]. For example, in conflict with the results of the present study, Zuo et al. had illustrated that miR-103a could be sensitive to mechanical loading [40], but, in the present study, myomiRs could not be a mechanosensitive miRNA following resistance training regimes in women with OSO. Finally, it was assumed that the threshold level of mechanical stress affecting skeletal muscle and bone remodeling had been affected by age, but the threshold values were not determined in the present study.

In accordance with the study hypothesis, some significant correlations were observed between changes in serum myomiRs and ALP, vitamin D, and FRAX® score following resistance training. Accordingly, it was hypothesized that myomiRs might induce osteogenic effects in response to 12 weeks of resistance training. Furthermore, myomiRs isolated from skeletal muscle samples might be involved in bone and muscle-associated health benefits. Recently, studies have suggested a possible role in cell-to-cell crosstalk, where myomiRs might be able to mediate gene expression in target tissues in a way comparable to hormones and myokines [41].

MyomiRs can be also crucial for bone health benefits conferred by exercise training modalities as they are responsible for cell-cell communication [42]. The miRNAs chosen a priori in this study are muscle-enriched ones previously described to regulate important genes in pathways central for skeletal muscle and bone tissues [43], which made them attractive candidates as exercise factors. For instance, miRNA-133a-5p inhibits the expression of osteoblast differentiation-associated markers by targeting the RUNX2 in bone marrow [44]. In addition, Ramos et al. had explained that high-intensity training had increased miR-133a and had consequently reduced skeletal muscle miRNA-133a [45].

The data from this study illustrated that miR-206 was the only myomiR with a negative correlation with skeletal muscle miR-133 expression in the control group. But, one recent study by Ultimo et al. had proven that endurance training could simultaneously modify miR-133a and miR-206 gene expression [46].

It should be noted that there were some limitations in the present study. First, the methodology utilized in this study allowed for analysis of only a few expressed myomiRs and osteoporosis markers and no other genome-wide approaches could be used (e.g., sequencing and microarrays). Secondly, the short duration of the given training protocol and its intensity might be reasons for the non-significant effect of exercise on some markers or lack of the moderator effect of myomiR status.

## Conclusion

Overall, the study findings uncovered the potential contribution of some bone metabolism markers in attenuating bone dysfunction during exercise modality, which could occur through some myomiRs regulating gene networks involved in bone remodeling. Furthermore, it was concluded that individual myomiRs engaged in myoblast and osteoblast differentiation might not regulate these myogenic and osteogenic targets in response to this type of exercise treatment i.e. resistance training.

## Supplementary Information

**Additional file 1: Table S1.** Elastic band resistance training protocol. **Table S2.** Elastic band resistance training protocol.

## Data Availability

All the data generated or analyzed during the present study were included in this paper.

## Abbreviations

BFP: Body fat percentage
OSO: Osteosarcopenic obesity
ICNs: Interorgan communication networks
myomiRs: Myotube-derived exosomal microRNAs
CTX-I: C-terminal telopeptides of type I collagen
FRAX: Fracture Risk Assessment Tool
ALP: Alkaline phosphatase
RCT: Randomized controlled trial
CONSORT: Consolidated Standards of Reporting Trials
DEXA: Dual-energy X-ray absorptiometry
BMI: Body mass index
TF: Total femur
FN: Femoral neck
10MWT: 10-m walk test
SMI: Skeletal muscle mass index
MoCA: Montreal Cognitive Assessment
CVDs: cardiovascular diseases
ANOV: Aanalysis of variance (ANOVA)
