# Supplementary material for: Effect of 12-weeks elastic band resistance training on MyomiRs and osteoporosis markers in elderly women with Osteosarcopenic obesity: a randomized controlled trial
Source: BMC Geriatr. 2021 Jul 20;21:433. doi: 10.1186/s12877-021-02374-9 (PMC8290586; doi:10.1186/s12877-021-02374-9)
Supplement: Supplementary file 1 — Additional file 1: Table S1. Elastic band resistance training protocol. Table S2. Elastic band resistance training protocol. [file 12877_2021_2374_MOESM1_ESM.docx]

Supplementary Table S1: Elastic band resistance training protocol

| **Movement** | **Intensity**  **(repetition/set)** | **Targeted muscle group** | **Duration (min)** |
| --- | --- | --- | --- |
| **A. Warm-up** |  |  |  |
| Mobility exercise of the neck, upper limbs, and back |  | Upper quarter flexors and extensors | 5 |
| Global flexion-extension of the lower limb |  | Lower quarter flexors and extensors | 5 |
| **B. Upper quarter** |  |  |  |
| 1.Seated chest press | 10-12 | Upper quarter extensors | 5-10 |
| 2.Seated row | 10-12 | Upper quarter flexors | 5-10 |
| 3.Seated elbow flexion | 10-12 | Elbow flexors | 5-10 |
| 4.Standing elbow extension | 10-12 | Elbow extensors | 5-10 |
| 5.Standing cable lateral raise | 10-12 | Upper quarter flexors | 5-10 |
| 6.Abdominal crunch with machine | 10-12 | Core muscles | 5-10 |
| **C. Lower quarter** |  |  |  |
| 7. Prone knee extension | 10-12 | Lower quarter extensors | 5-10 |
| 8. Seated leg curl | 10-12 | Lower quarter flexors | 5-10 |
| 9. Seated calf raise | 10-12 | Lower quarter extensors | 5-10 |
| 10. Seated leg abduction | 10-12 | Lower quarter flexors | 5-10 |
| 11. Seated leg adduction | 10-12 | Lower quarter flexors | 5-10 |
| 12. Seated knee extension | 10-12 | Hamstring groups | 5-10 |
| 13. Seated dorsiflexion | 10-12 | Ankle flexors | 5-10 |
| 14. Seated plantar flexion | 10-12 | Ankle plantar | 5-10 |
| **D. Cool down** |  |  | 5 |

The participants were instructed to complete the concentric phase for 3 s and perform the eccentric phase of each repetition over 7 s.

Supplementary Table S2: Elastic band resistance training protocol

|  | Week | 1,2 | 3,4 | 5,6 | 7,8 | 9,10 | 11,12 |
| --- | --- | --- | --- | --- | --- | --- | --- |
| Elastic band color | Yellow | X | X |  |  |  |  |
|  | Red |  |  | X | X |  |  |
|  | Green |  |  |  |  | X | X |
|  | Blue |  |  |  |  |  |  |
|  | Black |  |  |  |  |  |  |
|  | Silver |  |  |  |  |  |  |
| Exercise loading | Repetition | 10–12 | 10–12 | 10–12 | 10–12 | 10–12 | 10–12 |
|  | Set | 1 | 1 | 2 | 2 | 2 | 2 |
|  | RPE^a^ | 10–13 | 10–13 | 10–13 | 10–13 | 10–13 | 10–13 |

X=Color of the elastic band
